# Supplementary material for: The interactome of intact mitochondria by cross-linking mass spectrometry provides evidence for coexisting respiratory supercomplexes
Source: Mol Cell Proteomics. 2017 Dec 8;17(2):216–32. doi: 10.1074/mcp.RA117.000470 (PMC5795388; doi:10.1074/mcp.RA117.000470)
Supplement: Supplemental Data [file supp_RA117.000470_133922_0_supp_23978_fzffwf.zip › spectra_annotation/mito_DR_spectra_annotation/24-1-3-1-62-1.pdf]

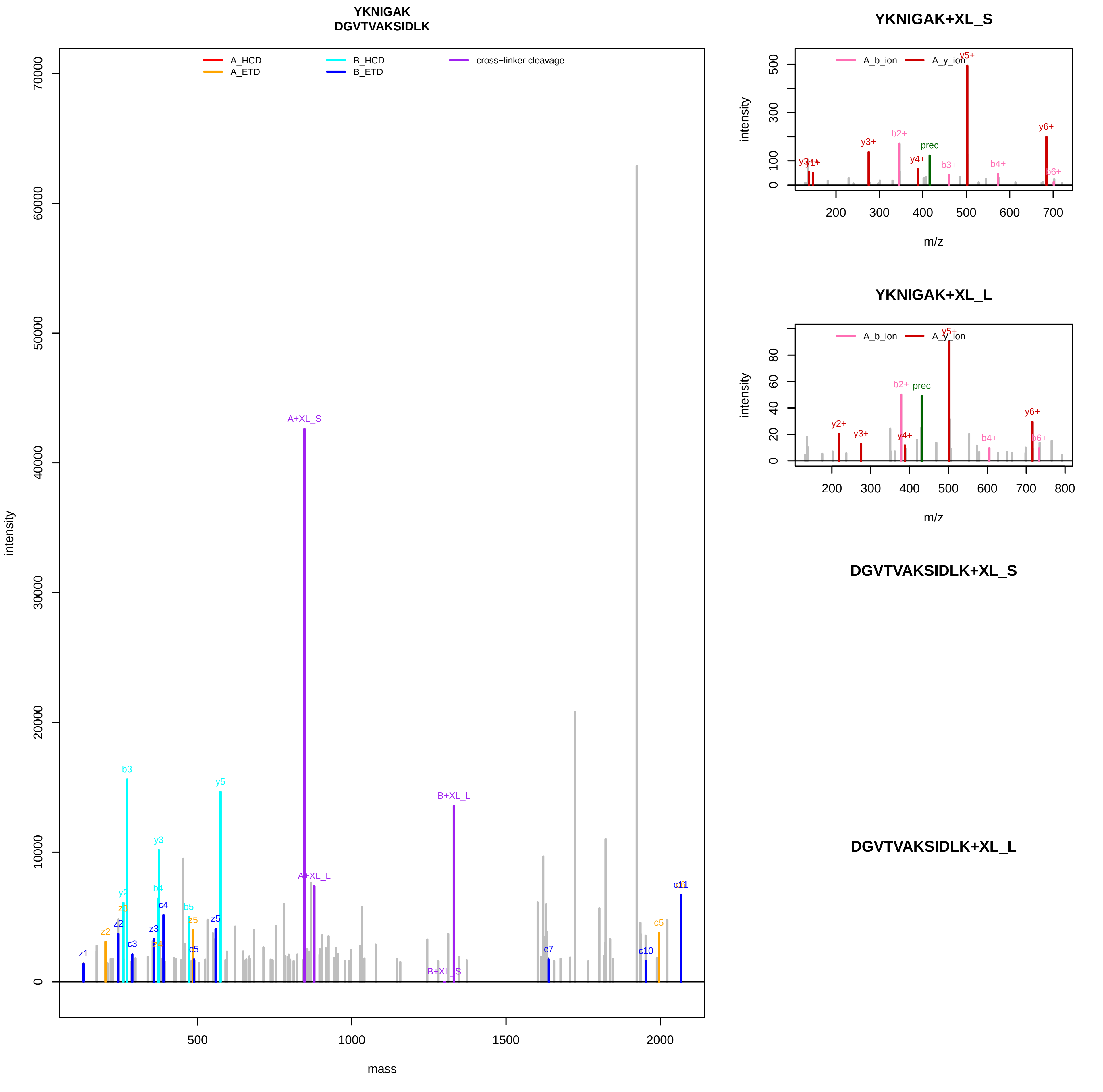
YKNIGAK
DGVTVAKSIDLK
A_HCD
A_ETD
B_HCD
B_ETD
cross−linker cleavage
A+XL_S
A+XL_L
B+XL_L
B+XL_S
b3
y5
y3
y2
b4
b5
c4
z3
z2
z5
c3
c5
z1
z2
z3
z4
z5
c7
c10
c5
c11
0
10000
20000
30000
40000
50000
60000
70000
500
1000
1500
2000
mass
YKNIGAK+XL_S
A_b_ion
A_y_ion
y5+
y3+
b2+
prec
y4+
b3+
b4+
y6+
b6+
m/z
YKNIGAK+XL_L
y2+
DGVTVAKSIDLK+XL_S
DGVTVAKSIDLK+XL_L
